# Septic Arthritis of the Temporomandibular Joint Complicated by Dislocation

**DOI:** 10.7759/cureus.105091

**Published:** 2026-03-12

**Authors:** Hideka Kanemoto, Kyoichi Obata, Yohei Takeshita, Koki Umemori, Soichiro Ibaragi

**Affiliations:** 1 Department of Oral Surgery, Kochi Health Sciences Center, Kochi, JPN; 2 Department of Oral and Maxillofacial Surgery, Okayama University Graduate School of Medicine, Okayama, JPN; 3 Department of Oral and Maxillofacial Radiology, Faculty of Medicine, Dentistry and Pharmaceutical Sciences, Okayama University, Okayama, JPN

**Keywords:** antibiotics, culture-negative infection, joint dislocation, septic arthritis, temporomandibular joint

## Abstract

Septic arthritis of the temporomandibular joint (SATMJ) is a rare but potentially serious condition, and standardized diagnostic and therapeutic strategies have not been established. We present the case of a 70-year-old man who developed acute right temporomandibular joint (TMJ) pain, swelling, mandibular deviation, and inability to achieve mouth closure. Computed tomography and magnetic resonance imaging revealed right TMJ dislocation, joint effusion, degenerative changes, and anterior disc displacement with effusion. Aspiration of the joint yielded neutrophil-predominant purulent fluid, although bacterial cultures were negative. The patient was treated empirically with intravenous ceftriaxone followed by oral clindamycin and amoxicillin, resulting in rapid symptom resolution, and the dislocation spontaneously reduced without surgical intervention. No recurrence was observed during three months of follow-up. This case highlights the diagnostic challenges associated with culture-negative SATMJ, supports the role of early empirical antibiotic therapy, and suggests that chronic joint instability due to habitual dislocation may predispose the TMJ to infection.

## Introduction

Septic arthritis of the temporomandibular joint (SATMJ) is a rare clinical condition. Due to its low incidence [1], there is no established consensus on its etiology or optimal management. SATMJ typically presents with preauricular pain, swelling, and trismus, and may involve the bone, cartilage, joint capsule, and surrounding musculature [2]. Although most cases are acute, chronic forms have also been documented [3]. The condition is generally linked to oropharyngeal, otologic, or odontogenic infections, or hematogenous dissemination [3]. In some cases, the primary source of infection remains unidentified. Diagnosis is based primarily on clinical presentation and imaging, with joint aspiration and culture playing a central role [4]. Nevertheless, culture-negative SATMJ poses significant diagnostic and therapeutic challenges [1]. Although standardized diagnostic criteria for SATMJ have not been universally established, the diagnosis is generally based on the combination of acute inflammatory symptoms, elevated inflammatory markers, imaging evidence of joint effusion, and supportive findings from joint aspiration [4]. This distinction is particularly important in culture-negative cases to differentiate SATMJ from noninfectious temporomandibular disorders.

SATMJ complicated by temporomandibular joint (TMJ) dislocation is exceedingly rare [1]. Chronic joint inflammation may lead to structural changes and capsular laxity, potentially contributing to dislocation, although the pathophysiology of this association remains unclear. We report a case of culture-negative SATMJ accompanied by TMJ dislocation in a 70-year-old male, noteworthy for the absence of an identifiable infection source despite concurrent acute septic arthritis and joint dislocation. This case provides new clinical insights into the pathogenesis and management of SATMJ.

## Case presentation

The patient, a 70-year-old male, initially visited a dental clinic with a chief complaint of right TMJ pain and was referred to our department for further evaluation. Initial examination revealed tenderness and swelling in the right preauricular region, with palpation confirming right TMJ dislocation, an inability to achieve occlusion, and mandibular deviation to the left.

**Figure 1:**
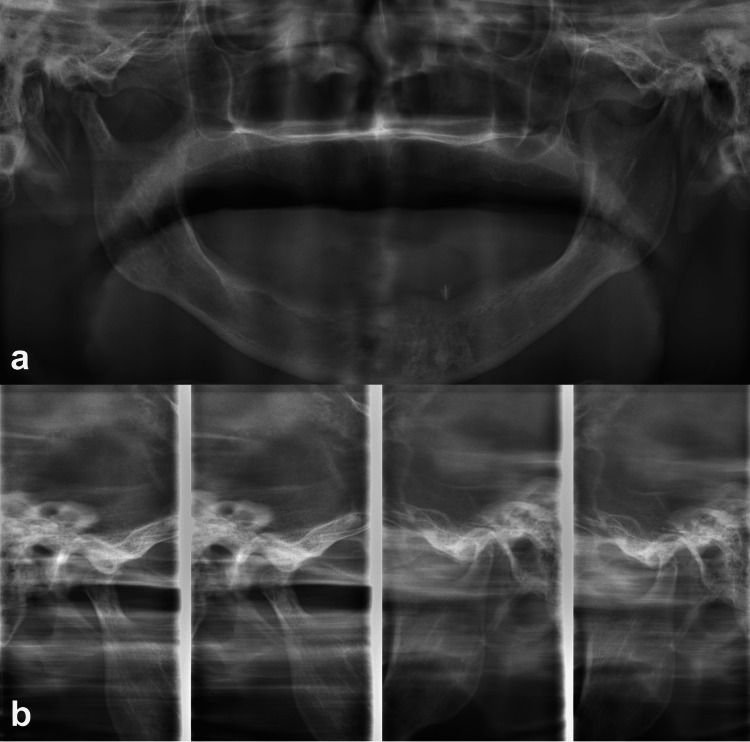
Panoramic image and temporomandibular joint mode image. (a) Panoramic image showing dislocation of the right temporomandibular joint, flattening of the mandibular condyle, and osteophyte formation. There was no finding of infection in the mandibular body and maxilla. (b) Temporomandibular joint mode image showing dislocation of the right temporomandibular joint to the anterior and inferior side, flattening of the mandibular condyle, and osteophyte formation.

**Figure 2:**
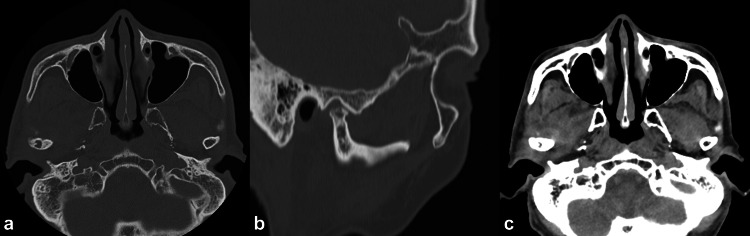
Computed tomography images. (a) Axial image of the bone showing coarse resorption of the right mandibular condyle with surrounding sclerosis. (b) Sagittal image of the bone showing coarse resorption of the right mandibular condyle with surrounding sclerosis and osteophyte formation. The articular fossa and eminence were relatively preserved. (c) Axial image of the soft tissue showing thickened soft tissue surrounding the right mandibular condyle.

Imaging, including a panoramic view (Figure 1a) and a TMJ mode image (Figure 1b), showed right TMJ dislocation, flattening of the mandibular condyle, and osteophyte formation. The only remaining tooth, the left mandibular canine, showed no pathological findings, and there was no evidence of odontogenic infection. A computed tomography (CT) scan revealed coarse resorption of the right mandibular condyle, with surrounding sclerosis and osteophyte formation, consistent with chronic degenerative changes (Figure 2a). The articular fossa and eminence appeared relatively preserved (Figure 2b), while soft tissue surrounding the right mandibular condyle was thickened (Figure 2c). These findings suggested long-standing joint instability or previously undiagnosed recurrent dislocations.

**Figure 3:**
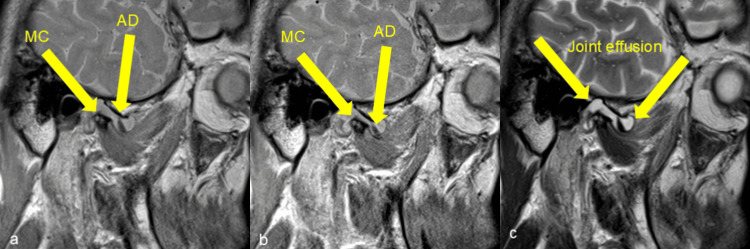
Magnetic resonance images of the right temporomandibular joint. (a) Sagittal proton density image during mouth closing. Anterior disc displacement was observed. (b) Sagittal proton density image during mouth opening. Anterior disc displacement without reduction was observed. (c) Sagittal T2-weighted image during mouth closing. A well-defined hyperintense region showing joint effusion was observed. MC: mandibular condyle; AD: articular disc

On magnetic resonance imaging (MRI), proton density images demonstrated anterior disc displacement without reduction on both sides (Figures 3a, 3b). T2-weighted images showed a well-defined, hyperintense joint effusion in the right TMJ, with no signs of abscess formation or adjacent tissue infiltration (Figure 3c).

An attempt to manually reduce the dislocated joint was unsuccessful due to severe pain. Therefore, reduction under general anesthesia with muscle relaxants was scheduled. Blood tests showed elevated C-reactive protein (CRP) at 16.22 mg/dL and procalcitonin (PCT) at 0.668 ng/mL, with no antibiotics administered at that time.

Under local anesthesia, yellow fluid was aspirated from a fluctuant area in the right cheek and sent for bacterial analysis. The patient was advised to be admitted immediately; however, hospitalization was deferred because of the patient’s personal circumstances.

The following day, spontaneous reduction of the right TMJ dislocation occurred before the planned procedure. Although the dislocation resolved, limited mouth opening and persistent swelling remained. Based on clinical findings and laboratory results, a diagnosis of septic arthritis of the right TMJ was made. The patient was admitted and treated with intravenous ceftriaxone (2 g/day). Improvement in pain and swelling was noted, and CRP levels decreased to 3.74 mg/dL.

On hospital day six, the patient was discharged in good condition and followed as an outpatient for three months, with no recurrence of symptoms. Surgical intervention was recommended to address the habitual TMJ dislocation, but the patient declined further treatment and did not return for follow-up.

## Discussion

SATMJ is a rare but potentially serious condition. Delayed diagnosis and treatment can lead to complications such as osteomyelitis, ankylosis, systemic infection [5], or even intracranial extension due to the anatomical proximity of the TMJ to critical structures [2]. In this case, differential diagnoses included acute exacerbation of internal derangement, osteoarthritis, rheumatoid arthritis, crystal-induced arthritis, and parotitis. These conditions were considered unlikely due to the marked elevation of inflammatory markers, presence of purulent aspirated fluid, and MRI findings demonstrating significant joint effusion. The overall clinical presentation was more consistent with septic arthritis. These complications can occur in any patient; however, in elderly or immunocompromised individuals, the disease may progress more rapidly, resulting in systemic manifestations such as sepsis or multi-organ failure [6]. In the present case, underlying capsular laxity due to habitual dislocation may have predisposed the TMJ to infection. SATMJ is considered extremely rare compared to septic arthritis of larger joints, likely due to the unique anatomical and clinical characteristics of the TMJ, including its limited joint space, relatively poor vascularization, and lower exposure to trauma or surgical intervention [7]. Hematogenous spread is generally required for septic arthritis to develop in the TMJ, often combined with local factors that compromise joint defenses, such as trauma, dislocation, intra-articular injection, or prior surgery [2]. This may partly explain the extreme rarity of SATMJ compared to septic arthritis of larger joints. The reported incidence of septic arthritis ranges from 4 to 29 cases per 100,000 person-years, with increased risk in older individuals, those receiving immunosuppressive therapy, and populations with lower socioeconomic status [8]. By 2022, only 33 cases of SATMJ had been documented [3]. Among these 33 cases, 21 were male, and 12 were female. A chi-squared analysis showed no statistically significant difference between sexes (p > 0.1), suggesting that SATMJ may affect both genders equally. In a review of these cases, *Staphylococcus aureus* was the most frequently identified pathogen (10 cases), including one patient with rheumatoid arthritis and three elderly individuals. *Proteus mirabilis* was reported in three cases [9-11], one of which involved mixed infection with *Staphylococcus aureus* and *Enterococcus*, while the remaining two had no clearly described risk factors. *Neisseria gonorrhoeae* was implicated in two cases [12,13], both associated with sexually transmitted infections. Other rare pathogens included *Haemophilus influenzae*, *Pseudomonas aeruginosa*, and *Clostridium* species [14-16]. In particular, *Pseudomonas* infections were thought to result from malignant otitis externa in diabetic patients [16].

Despite efforts to isolate causative organisms, culture testing often fails to identify a pathogen. In a retrospective review, 85.7% of SATMJ cases yielded negative culture results [1]. In the present case, the pale-yellow synovial fluid aspirated from the TMJ was also sterile on culture. Culture negativity may result from prior antibiotic therapy, effective host immune clearance, or insufficient bacterial load. The patient had received antibiotics for an unrelated condition approximately one week earlier, which may have contributed to culture negativity. Although the patient’s white blood cell count was normal, inflammatory markers such as CRP and PCT were significantly elevated, indicating ongoing infection. This case underscores the importance of considering septic arthritis even in the absence of positive cultures or leukocytosis. While this case suggests a potential association between habitual TMJ dislocation and septic arthritis, such a relationship is likely rare and should be interpreted with caution. This case highlights joint instability as a local risk factor that may predispose to infection, rather than suggesting a causal link. More importantly, this case underscores the need for prompt treatment of SATMJ due to its proximity to critical structures such as the infratemporal fossa and cranial base, as delayed intervention may lead to serious complications, such as intracranial spread and fibrous ankylosis [1,3]. Empirical antibiotic therapy should not be withheld in culture-negative cases, especially when clinical and imaging findings suggest infection. Broad-spectrum intravenous antibiotics with good synovial penetration are recommended in the acute phase of SATMJ, particularly in culture-negative cases [1]. In this case, ceftriaxone was selected as the initial empirical agent because of its broad-spectrum Gram-positive and Gram-negative coverage, established efficacy in septic arthritis, favorable synovial penetration, and convenient once-daily dosing. However, it lacks anaerobic coverage and is ineffective against β-lactamase-producing organisms. Recent studies suggest that empirical regimens for SATMJ often include a third-generation cephalosporin combined with metronidazole [17]. Given the frequent involvement of anaerobic bacteria and resistant Gram-positive cocci, β-lactam/β-lactamase inhibitor combinations such as ampicillin-sulbactam may provide broader and more appropriate coverage. Although ceftriaxone was effective in this case, the possibility of anaerobic or β-lactamase-producing bacterial involvement in SATMJ cannot be entirely excluded. Therefore, β-lactam/β-lactamase inhibitor combinations may represent a more comprehensive empirical option in future cases, particularly when the causative organism is unknown or mixed infection is suspected.

This case has several limitations. First, as this is a single case report, a causal relationship between chronic TMJ instability and septic arthritis cannot be established. It remains unclear whether habitual dislocation predisposed the joint to infection or whether acute infection contributed to joint dislocation. Second, despite clinical, radiologic, and laboratory findings strongly suggestive of septic arthritis, no pathogen was identified, which limits microbiological confirmation. Third, quantitative synovial fluid analysis and Gram stain results were not available. Finally, although no recurrence was observed during the three months of follow-up, longer-term observation would be necessary to better evaluate clinical outcomes. Future accumulation of similar cases is needed to clarify the pathophysiological relationship and optimize management strategies for SATMJ.

## Conclusions

SATMJ remains a diagnostic challenge, particularly when cultures are negative. This case underscores the importance of early empirical antibiotic therapy based on clinical and radiologic findings. Additionally, the presence of pre-existing joint instability due to habitual dislocation may serve as a potential risk factor for the development of SATMJ and should be carefully evaluated during diagnosis and treatment planning.

## References

[1] Cai XY, Yang C, Zhang ZY, Qiu WL, Chen MJ, Zhang SY (2010). Septic arthritis of the temporomandibular joint: a retrospective review of 40 cases. J Oral Maxillofac Surg.

[2] Gayle EA, Young SM, McKenna SJ, McNaughton CD (2013). Septic arthritis of the temporomandibular joint: case reports and review of the literature. J Emerg Med.

[3] Goldschmidt MJ, Butterfield KJ, Goracy ES, Goldberg MH (2002). Streptococcal infection of the temporomandibular joint of hematogenous origin: a case report and contemporary therapy. J Oral Maxillofac Surg.

[4] Araidy S, Maalouf N, Front E, Abu El-Naaj I (2024). Septic arthritis of the temporomandibular joint-a case report and review of the literature. Front Oral Health.

[5] Al-Khalisy HM, Nikiforov I, Mansoora Q, Goldman J, Cheriyath P (2015). Septic arthritis in the temporomandibular joint. N Am J Med Sci.

[6] Sun X, Li Y, Lv Y, Liu Y, Lai Z, Zeng Y, Zhang H (2024). Diagnostic value of procalcitonin in patients with periprosthetic joint infection: a diagnostic meta-analysis. Front Surg.

[7] Azmi NS, Mohamad N, Razali NA, Zamli AK, Sapiai NA (2021). Septic arthritis of temporomandibular joint. Med J Malaysia.

[8] Earwood JS, Walker TR, Sue GJ (2021). Septic arthritis: diagnosis and treatment. Am Fam Physician.

[9] Stoelinga PJ, Gallia L, Tideman H, Soudijn ER (1980). [Chronic iatrogenic arthritis of the temporomandibular joint]. Fortschr Kiefer Gesichtschir.

[10] Seymour RA, Summersgill GB (1982). Haemophilus influenzae pyarthrosis in a young adult with subsequent temporomandibular joint involvement. Br J Oral Surg.

[11] Goodman WS, Strelzow VV (1979). Infections of the temporomandibular joint. J Otolaryngol.

[12] Alexander WN, Nagy WW (1973). Gonococcal arthritis of the temporomandibular joint. Report of a case. Oral Surg Oral Med Oral Pathol.

[13] Chue PW (1975). Gonococcal arthritis of the temporomandibular joint. Oral Surg Oral Med Oral Pathol.

[14] Takaku S, Ozawa S, Ueda Y, Yoshimoto T (1981). Acute suppurative arthritis of the temporomandibular joint: report of a case. Jpn J Oral Surg.

[15] Matsumura Y, Inui M, Tagawa T (1998). Peritemporomandibular abscess as a complication of acupuncture: a case report. J Oral Maxillofac Surg.

[16] Midwinter KI, Gill KS, Spencer JA, Fraser ID (1999). Osteomyelitis of the temporomandibular joint in patients with malignant otitis externa. J Laryngol Otol.

[17] Jovanović M, Milosavljević M, Zdravković D, Živić M, Veličković S, Janković S (2022). Septic arthritis of the temporomandibular joint in adults: systematic review. J Stomatol Oral Maxillofac Surg.

